# Transcriptomic and Metabolomic Insights into Growth Performance of a Novel Allotriploid Fish Derived from *Carassius cuvieri*, *Carassius auratus* red var., and *Cyprinus carpio*

**DOI:** 10.3390/ani16152341

**Published:** 2026-08-01

**Authors:** Fanglei Liu, Huirong Yang, Hongwen Liu, Ziyi Huang, Xuanyi Zhang, Bei Li, Lujiao Duan, Jianming Yu, Siyang Huang, Qizhi Liu, Min Tao, Chun Zhang, Qingfeng Liu, Shaojun Liu

**Affiliations:** 1 College of Marine Sciences, South China Agricultural University, Guangzhou 510642, China; fangleiliu2026@163.com (F.L.); hry@scau.edu.cn (H.Y.); 2 Engineering Research Center of Polyploid Fish Reproduction and Breeding of the State Education Ministry, College of Life Sciences, Hunan Normal University, Changsha 410081, China; liuhongwen2002@163.com (H.L.); 17369243016@163.com (Z.H.); zhangxuanyi@xhlab.ac.cn (X.Z.); 17321063030@163.com (B.L.); 15307344849@163.com (L.D.); 1060929506a@gmail.com (J.Y.); 18874815295@163.com (S.H.); lqz@hunnu.edu.cn (Q.L.); minmindiu@126.com (M.T.); zhangchun421@163.com (C.Z.)

**Keywords:** triploid fish, growth characteristics, metabolomics, transcriptomics

## Abstract

Triploid fish have attracted considerable attention in aquaculture because of their sterility and rapid growth. In this study, a novel allotriploid fish (WA, 3*n* = 150) was obtained through distant hybridization of a female diploid hybrid fish (WR-II, 2*n* = 100) with a male allotetraploid fish (4nAT, 4*n* = 200). The measurable traits of WA exhibit significant differences from WR-II and its original parents (white crucian carp (WCC) and red crucian carp (RCC)). WA muscles exhibited higher protein, fat, and essential amino acid levels but lower moisture. Furthermore, WA enhanced growth performance and muscle hypertrophy compared to WCC and RCC. The transcriptomes and metabolomics of the muscle tissues of WA, WCC, and RCC were analyzed. In total, 3889 and 1863 differential expressed genes (DEGs) were identified in WA vs. WCC and WA vs. RCC, respectively. These DEGs were mainly enriched in pathways related to amino acid biosynthesis and oxidative phosphorylation. In total, 421 and 304 differential metabolites (DMs) were identified in WA vs. WCC and WA vs. RCC, respectively. These DMs were mainly enriched in the pathways related to glycerophospholipid and fatty acid metabolism. Integrated transcriptomic and metabolomic analyses revealed that the expression patterns of *acta1*, *myh*, *myog*, and *aldoa* were associated with changes in the abundance of oleoyl ethanolamide, betaine, inosine, docosahexaenoic acid (DHA), and PC (38:6), suggesting their potential involvement in the enhanced growth performance of WA. This study provides a potential genetic resource for aquaculture and identifies molecular features associated with enhanced growth performance in this allotriploid fish.

## Simple Summary

Triploid fish have attracted considerable attention in aquaculture because of their sterility and, in some cases, enhanced growth performance. This study successfully generated a novel allotriploid fish (WA, 3*n* = 150) through distant hybridization between female diploid hybrid fish and male allopolyploid fish. This hybrid combination exhibited high fertilization rates (89.22%) and high hatching rates (78.71%), indicating potential for efficient fry production. WA demonstrated higher levels of protein, fat, and essential amino acids in their muscle tissue. Furthermore, compared to control fish, WA exhibits accelerated growth and muscle hypertrophy. Integrated transcriptomic and metabolomic analyses revealed that the expression patterns of *acta1*, *myh*, *myog*, and *aldoa* were associated with changes in the abundance of oleoyl ethanolamide, betaine, inosine, docosahexaenoic acid, and PC (38:6). Changes in the abundance of growth-related genes and metabolites showed a significant co-expression trend in WA muscle, suggesting that these molecular changes may be associated with the enhanced growth performance of WA.

## 1. Introduction

Distant hybridization is a breeding strategy that combines genetic materials from different species or lineages and may generate substantial genomic and phenotypic variation in offspring. In polyploid hybrids, genome elimination, rearrangement, and chromosome loss may also occur [1]. The hybrid progeny often displays heterosis, especially in traits such as growth and disease resistance. Enhanced growth in triploid fish may result from multiple factors, including heterosis, polyploidization-associated changes in cell size and metabolism, altered energy allocation due to sterility, hormonal regulation, and selective breeding [2]. Many advantageous triploid fish were obtained through distant hybridization, such as through female blunt snout bream (*Megalobrama amblycephala*) × male Bleeker’s yellow tail (*Xenocypris davidi* Bleeker) to obtain fast-growing triploid fish [3]; hybrids obtained by female WCC (*Carassius cuvieri*) × male allotetraploid were characterized by fast growth and high resistance [4]; hybrid progeny obtained using female RCC (*Carassius auratus* red var.) × male rare gudgeon (*Gobiocypris rarus*) were also characterized by fast growth [5]. Owing to these advantageous traits, triploid fish, particularly salmonids, have been widely applied in aquaculture mainly because of their sterility and potential advantages in growth performance and production management [6,7]. Although hybridization has been utilized for breeding innovation in a wide range of fish species, not all hybridizations result in beneficial phenotypes. Hybridization can disrupt co-adapted gene complexes in fish, leading to phenomena such as outbreeding depression, reduced survival rates, and decreased fertility in the F2 generation or subsequent generations [8,9,10,11,12]. At the ecological level, fast-growing hybrid fish may outcompete native fish species [13,14,15,16]. For example, in European waters, the hybridization rate between native crucian carp and non-native crucian carp or common carp is as high as 82% [17]. Triploid fish are typically sterile; this characteristic not only reduces unnecessary sexual maturation during aquaculture but also eliminates the risk of escaped individuals exerting a negative genetic influence on wild populations [18,19,20]. Therefore, we believe that distant hybridization provides a pivotal strategy for generating novel fish with enhanced commercial value and that these fish provide valuable materials for studying important economic traits.

In studying economically important traits in fish, multi-omics integration analysis has become a crucial method for clarifying traits, such as rapid growth and cold tolerance [21]. Integrated multi-omics analysis can provide insights into molecular pathways and candidate regulatory factors associated with complex traits. For example, by integrating metabolomics and transcriptomics, changes in the liver metabolites of fish under low-temperature stress have been analyzed in various metabolic pathways [22]. By combining microbiome and metabolomic data, the gut flora and metabolites in herbivorous fish have been shown to exhibit coexistence and differentiation [23]. Key genes and metabolites for rapid growth in hybrid fish were identified by integrating 16s rRNA, metabolomics, and transcriptomics [24]. Therefore, integrated multi-omics analysis can reveal the molecular mechanisms of trait formation and provide a scientific basis for the molecular breeding of fish and the cultivation of superior varieties.

We previously obtained hybrid strain WR (2*n* = 100, F_1_–F_5_) by crossbreeding female WCC (2*n* = 100) with male RCC (2*n* = 100). Through backcrossing female WR-F_1_ and male WCC, we produced a new and improved fish, WR-II (2*n* = 100) [25]. WR-II is a diploid fish with a smaller head, higher body weight, and faster growth rate than its parents. In addition, an allotetraploid strain (4nAT, 4*n* = 200, F_3_–F_23_) was obtained by crossing female RCC with male common carp (*Cyprinus carpio*, 2*n* = 100) [1]. Both WR-II and 4nAT are high-quality and important germplasm resources, and triploid offspring with hybrid vigor can be produced by crossing WR-II and 4nAT.

In this study, we successfully generated a novel allotriploid fish (WA) via distant hybridization between diploid female WR-II and allotetraploid male 4nAT. The biological characteristics of WA, including ploidy, chromosome number, morphological characteristics, muscle nutrition, and growth rate, were analyzed using muscle metabolomics and transcriptomics, which revealed crucial genes and metabolites associated with its growth. We hypothesized that the enhanced growth performance of triploid WA is associated with changes in energy metabolism and protein synthesis pathways. This study provides new fish germplasm for aquaculture and a reference for understanding molecular features associated with rapid growth in this allotriploid fish.

## 2. Materials and Methods

### 2.1. Creation, Breeding, and Morphological Measurement of Hybrid Offspring WA

The experimental fish, including WR-II, 4nAT, WCC, and RCC, were sourced from the Yuelushan Laboratory Aquatic Variety Breeding Center, Hunan Normal University, China. During the 2023 breeding season (March to June), 60 sexually mature one-year-old female WR-II (500–600 g each) and 40 one-year-old male 4nAT (400–500 g each) were selected for artificial spawning. Female WR-II were encouraged to produce eggs using hormones (HCG: 1000 IU/kg, A2: 10 µg/kg, DOM: 0.02 mL/kg), whereas male 4nAT did not require hormone treatment. Artificial fertilization was performed following established protocols [26], yielding hybrid offspring designated as WA. Mature oocytes and semen were collected separately into dry containers. Approximately 0.5 mL of sperm was mixed with approximately 20 g of eggs, and fertilization was initiated immediately by adding 200 mL of aerated tap water (22–24 °C). The embryos were incubated in Petri dishes with water temperature maintained at 22–24 °C. A random sample of 10,000 embryos was selected from the embryo population to calculate both the fertilization rate (number of fertilized eggs past the proto-gastrula stage/total number of fertilized eggs × 100%) and hatching rate (number of fry/total number of fertilized eggs × 100%). In addition, 10 mature females and 10 mature males each of WCC, RCC, and 4nAT were selected for selfing to obtain their respective selfed offspring. Meanwhile, 10 mature WR females were crossed with 10 mature WCC males to produce WR-II offspring. After hatching, 200 individuals each of WA, RCC, and WCC (total 600 fish) were reared together in the same pond (200 m^2^). Additionally, 300 individuals each of WR-II and 4nAT (total 600 fish) were raised together in another pond (200 m^2^). The two ponds were adjacent, with identical areas and volumes. Both ponds maintained dissolved oxygen levels >5 mg/L, water temperatures between 19 and 25 °C, and pH values ranging from 7 to 8.5. At 12 months of age, 100 individuals from each of the WA, WCC, RCC, WR-II, and 4nAT populations (total 500 fish) were randomly selected for morphological measurement and analysis.

### 2.2. Detection of the Ploidy Level

The DNA content in erythrocytes of 1-year-old WR-II, 4nAT, and WA (50 fish per group) was measured by flow cytometry. Blood samples were collected from the caudal vein using a syringe pre-coated with a small amount of sodium heparin (ACD). Samples were labeled and kept on ice until analysis. For DNA staining, 1–3 µL of whole blood was added to 300 µL of DAPI staining solution and incubated at room temperature in the dark for 15 min. The DNA content was subsequently measured using a flow cytometer [27]. The χ^2^ test with Yates’ correction was applied to compare DNA content ratios between WA and WR-II. Kidney cell suspensions from 10 one-year-old WA fish were prepared for chromosome analysis [28]. Head and kidney tissues were homogenized in 0.01% colchicine RPMI 1640 medium, incubated at 37 °C for 2 h, subjected to a 0.075 M KCl hypotonic treatment for 30 min, fixed three times with Carnoy’s fixative (methanol: glacial acetic acid = 3:1), and stained with Giemsa following mounting. A total of 100 metaphase spreads (10 per fish) were examined under a light microscope to determine chromosome numbers.

### 2.3. Muscle Composition, Amino Acid Content, Weight Statistics, and Muscle Fiber Analysis

Fish weights were counted at 2-monthly intervals, and 100 randomly selected, lightly anesthetized fish of each group (WA, RCC, and WCC) were weighed and recorded. Two key growth indices were evaluated: weight gain rate (WGR, %) and specific growth rate (SGR, %/day). These values were calculated using the following formula: WGR (%) = 100 × (Waf − Wai)/Wai and SGR (%/d) = 100 × [ln(Waf) − ln(Wai)]/d, where Waf and Wai (Wai = 5.20 ± 0.40 g) represent the average weights at 12 months and at initial weight (g), respectively, and d represents the number of days between initial and final weight measurements (d = 360). Wai represents the measurements taken for all experimental fish at the same age (30 days after hatching). All fish were fasted for 24 h prior to measurement to eliminate the effect of feeding on body weight. Statistical analyses were performed on 30 muscle cross-sections from the WA, WCC, and RCC groups. The method of muscle fiber analysis referred to a previous report [5]. Thirty fish were randomly selected from each strain (WA, WCC, RCC) (90 fish in total), with only one muscle sample taken from each fish. The sampling sites were fixed as the white muscle above the lateral line and just below the dorsal fin. The samples were fixed in 4% paraformaldehyde for 24 h, dehydrated in a series of ethanol solutions, embedded in paraffin, and sectioned transversely to a thickness of 5 μm. The sections were then stained with hematoxylin and eosin (H&E). Additionally, the moisture, protein, fat, ash, and amino acid contents of dorsal muscle samples from WA, WCC, and RCC were determined. Ten fish were randomly selected from each strain (WA, WCC, RCC) to serve as biological replicates (*n* = 10). Prior to sampling, all fish were fasted for 24 h to empty their intestinal contents. Each fish was dissected individually, and white muscle was harvested from both sides of the back. Approximately 5 g of muscle was collected from each fish, rapidly frozen in liquid nitrogen, and stored at −80 °C. Samples were not pooled; each fish was analyzed separately. The moisture content was measured by vacuum freeze-drying [29]; protein by the Kjeldahl method; fat by Soxhlet extraction [30]; and ash by combustion at 550 °C [31]. Amino acids were analyzed by acid hydrolysis followed by HPLC (Agilent 1200 series, Agilent Technologies, Santa Clara, CA, USA). The carbohydrate content was determined by the difference method [32].

### 2.4. Muscle Metabolomics Analysis

We conducted a metabolomic analysis of 18 muscle samples (6 WA, 6 WCC, and 6 RCC) using untargeted liquid chromatography-mass spectrometry (LC-MS). LC-MS analysis was performed according to established methods [33], with chromatographic separation conducted as described previously (Table S1) [34]. Data acquisition was carried out in both positive and negative ion modes, and the resulting raw files were processed using CD 3.3 software. The metabolites were identified and quantified by matching them against the mzCloud, mzVault, and MassList databases. Differential metabolites (DMs) were selected based on the following criteria: variable importance in projection (VIP) > 1.0, fold change (FC) > 1.5 or <0.667, and statistical significance (*p* < 0.05). Metabolite identification was performed using the Kyoto Encyclopedia of Genes and Genomes (KEGG), Human Metabolome Database (HMDB), and Lipid Metabolites and Pathways Strategy (LIPID MAPS) databases.

### 2.5. Analysis of Muscle Transcriptomics

Muscle transcriptomes from nine samples (3 WA/3 WCC/3 RCC) were sequenced. Total RNA was extracted from the muscle tissue using an RNA extraction kit (Omega, Miami, FL, USA) and subsequently used for library preparation and sequencing. All RNA samples had RIN values of ≥7.0. The sequencing platform used was the Illumina HiSeq 4000. Clean reads were aligned to the RCC genome (GCA_009069565.1) for comparative analysis, and differentially expressed genes (DEGs) were identified using the DESeq2 R package with thresholds of padj < 0.01 and |log2FC| > 1.5 in both the WA vs. WCC and WA vs. RCC comparisons. Growth-related genes were further characterized using Gene Ontology (GO) and KEGG pathway analyses.

### 2.6. Combined Metabolome and Transcriptome Analysis

We constructed two-way orthogonal partial least square (O2PLS) models using two comparative datasets, WA vs. WCC and WA vs. RCC, incorporating both DMs (VIP > 1 and *p* < 0.05) and DEGs (padj < 0.01 and |log2FC| > 1.5) as input data. The five candidate genes and five metabolites with the highest contributions to the O2PLS model were selected for further analysis. The selected DMs and DEGs were then mapped to the KEGG pathway database to elucidate their involvement in major biochemical and signaling pathways.

### 2.7. Real-Time Quantitative PCR

Total RNA was extracted from the WA, WCC, RCC, WR-II, and 4nAT muscle samples using a commercial RNA isolation kit (Omega, USA), followed by cDNA synthesis using a reverse transcription kit (Thermo Fisher Scientific, Waltham, MA, USA). Primers were designed based on conserved sequences shared among WCC, RCC, and *Cyprinus carpio* (Table S2) for triplicate gene expression analysis, with β-actin serving as the internal gene. Primers were designed within these conservative regions to ensure that they exhibit the same binding efficiency and amplification characteristics across samples from three different genetic backgrounds. All primers were verified by BLAST (https://blast.ncbi.nlm.nih.gov; accessed on 10 November 2025) to have no non-specific matches. A standard curve was constructed using 5-fold serial dilutions of cDNA (five dilution levels); the E-values for all primers fell within the range of 90–110% (R^2^ > 0.99). Melting curve analysis was performed after each reaction cycle to confirm the presence of a single peak. Quantitative real-time PCR was conducted on a Quant Studio 5 system (Life Technologies, Carlsbad, CA, USA) using PowerUp SYBR Green Master Mix (Thermo Fisher Scientific). Calculation of the relative gene expression levels was performed using the 2^−ΔΔCT^ method.

### 2.8. Statistical Analysis of Data

All data are expressed as the mean ± standard error (S.E.). Independent *t*-tests were performed using SPSS (version 26.0; IBM, Armonk, NY, USA). One-way analysis of variance (ANOVA) was used for comparisons between multiple groups. Prior to conducting an ANOVA, the Shapiro–Wilk test was used to assess the normality of the data, and Levene’s test was used to assess homogeneity of variances. Where the ANOVA was significant (*p* < 0.05), Tukey’s HSD test was used for post-hoc multiple comparisons. All analyses were performed in R 4.2.1. The calculation of the Pearson correlation coefficient assumes that there is a linear relationship between the two variables and that the residuals follow a normal distribution. Statistical analyses and graph generation were carried out with GraphPad Prism 10 (GraphPad Software, Boston, MA, USA). Relationships among multiple datasets were assessed using Pearson’s correlation analysis. Statistical significance was set at *p* < 0.05.

## 3. Results

### 3.1. The Formation and Morphological Characteristics of WA

**Figure 1:**
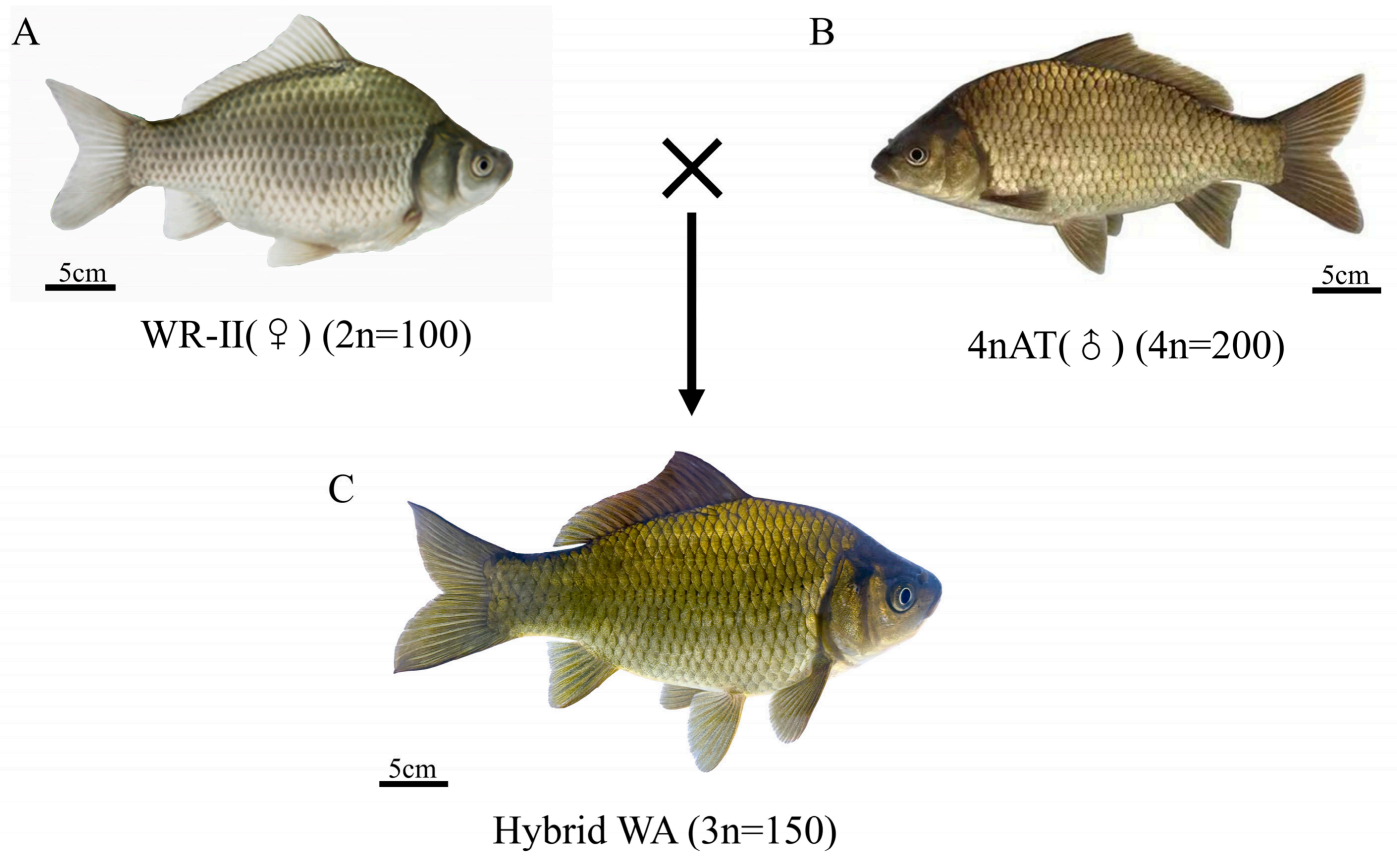
The crossing procedure and phenotypes of WA and its parents. (**A**) Appearance of WR-II. (**B**) Appearance of 4nAT. (**C**) Appearance of WA.

The fertilization and hatching rates of WA were 89.22% and 78.71%, respectively (Table S3). The creation process and phenotypic features of WA are shown in Figure 1. The measurable and countable traits of WA, WCC, RCC, WR-II, and 4nAT are shown in Table 1 and Table 2. The measurable traits of WA differed significantly from those of WR-II and its parents (WCC and RCC). Among the measurable traits (Table 1), BL/BH and CPL/CPH of WA were significantly greater than those of maternal WR-II. The countable traits of WA did not differ significantly from those of the parent strains (Table 2).

### 3.2. The Ploidy and Chromosome Number of WA

**Figure 2:**
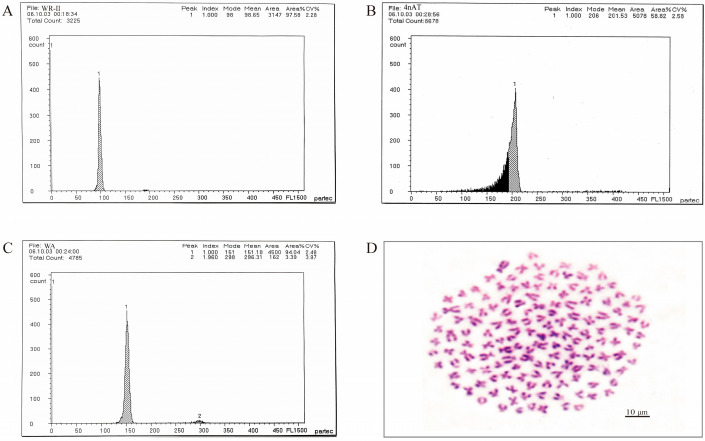
Cytometric histograms of DNA fluorescence and for WR-II, 4nAT, and WA, and the metaphase chromosome spreads of WA. (**A**) The mean DNA content of WR-II (peak 1: 98.65). (**B**) The mean DNA content of 4nAT (peak 1: 201.53). (**C**) The mean DNA content of WA (peak 1: 151.18). (**D**) The metaphase chromosome spreads of WA (3*n* = 150).

Flow cytometric analysis showed that the ratio of DNA content between WA and WR-II was 1.53 (151.18/98.65), using the DNA content of WR-II and 4nAT as a reference control (Figure 2A–C) (Table S5). Analysis of 100 metaphase spreads confirmed that WA contained the expected number of 150 chromosomes (Figure 2D, Table S5). These results confirmed that WA is a triploid fish with 150 chromosomes.

### 3.3. Muscle Composition and Amino Acid Content of WA

Comparative analysis of muscle composition revealed significant differences among WA, WCC, and RCC (Table 3 and Table 4). WA muscle exhibited lower moisture and carbohydrate contents than both WCC and RCC, while demonstrating higher fat and protein contents, as well as total energy value. In terms of amino acid composition, the contents of total flavor-related amino acids (DAA), total essential amino acids (EAA), and total amino acids (TAA) in WA muscle (5.36%, 5.89%, and 14.25%, respectively) were significantly higher than those in WCC (4.06%, 4.52%, and 10.89%, respectively) and RCC (4.67%, 5.22%, and 12.59%, respectively) (*p* < 0.05).

### 3.4. Growth Performance and Muscle Fiber Characteristics of WA

**Figure 3:**
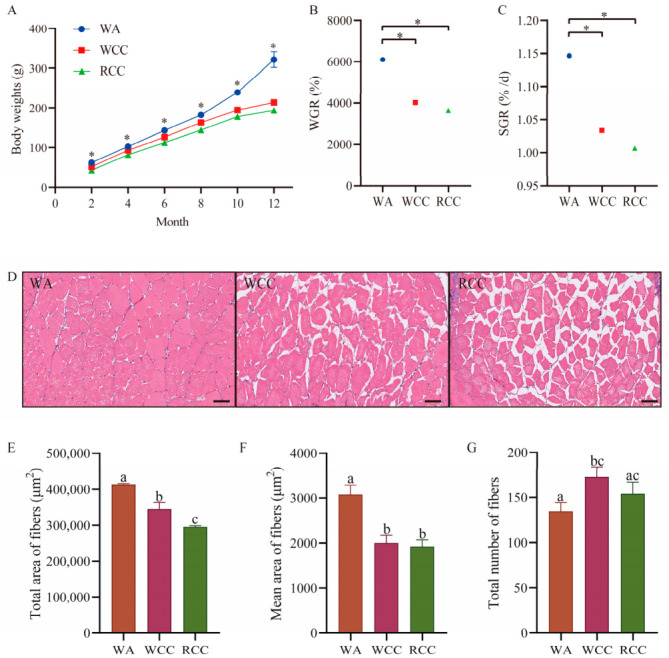
The average body weight of triploid WA was higher than that of WCC and RCC, and muscle fibers showed hypertrophy. (**A**) Average body weights of WA, WCC, and RCC from 2 to 12 months of age. (**B**) Weight gain rate for WA, WCC, and RCC. (**C**) Specific growth rates for WA, WCC, and RCC. (**D**) Paraffin sections and H&E staining of 12-month-old WA, WCC, and RCC. Bar = 50 μm. (**E**–**G**) Quantitative analysis of muscle fibers from WA, WCC, and RCC using ImageJ (v1.54q; National Institutes of Health, Bethesda, MD, USA) included calculating the total area of fibers, mean area of fibers, and total number of fibers. * and different lowercase letters indicate significant differences (*p* < 0.05).

WA exhibited a significantly higher mean body weight at all measured ages than the WCC and RCC groups (Figure 3A). The WGR (6100.13%) and SGR (1.15%/d) of WA were significantly higher than those of WCC (4023.13%, 1.03%/d) and RCC (3643.75%, 1.00%/d) (Figure 3B,C) (Table S6). Muscle fiber analysis revealed that WA exhibited muscle hypertrophy, with a significantly larger total fiber area (413,626.77 ± 1974.62 µm^2^) than WCC (344,528.93 ± 17,672.49 µm^2^) and RCC (295,230.20 ± 2829.23 µm^2^) (Figure 3E). However, WA had fewer total fiber numbers (134.67 ± 9.45) than WCC (172.67 ± 11.15) (Figure 3F). Additionally, the mean fiber area of WA (3080.67 ± 212.26 µm^2^) was significantly greater than that of both WCC (2001.00 ± 168.40 µm^2^) and RCC (1925.00 ± 144.56 µm^2^) (Figure 3G).

### 3.5. Muscle Metabolomics Analysis

**Figure 4:**
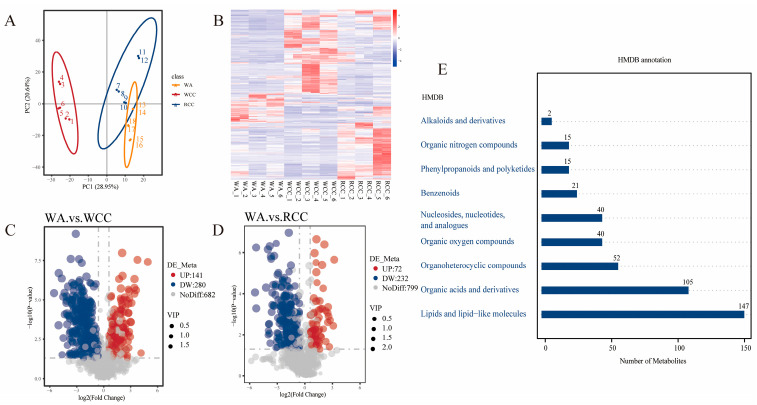

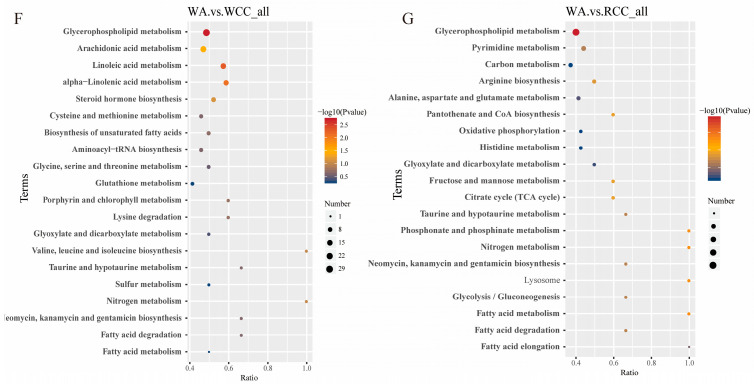
Identification and analysis of differentially metabolites in WA, WCC, and RCC. (**A**) PCA plot. (**B**) Differential abundant metabolite clustering heatmap; the vertical direction represents the clustering of samples, and the horizontal direction represents the clustering of metabolites. (**C**,**D**) Differential metabolite volcano map. (**E**) Differentially abundant metabolite HMDB classification statistical chart. (**F**,**G**) Differentially abundant metabolite KEGG enrichment map.

LC-MS analysis of muscle metabolites in WA, WCC, and RCC (Figure 4) identified 421 significant DMs between WA and WCC (141 upregulated, 280 downregulated; Figure 4C) and 304 between WA and RCC (72 upregulated, 232 downregulated; Figure 4D). HMDB classification showed that these differential DMs were primarily lipids and lipid-like molecules, organic acids and derivatives, and organoheterocyclic compounds (Figure 4E). KEGG enrichment analysis revealed distinct metabolic profiles between the groups; WA vs. WCC showed significant enrichment in glycerophospholipid metabolism, nitrogen metabolism, steroid hormone biosynthesis, and fatty acid metabolism (Figure 4F). In contrast, WA vs. RCC exhibited enrichment in glycerophospholipid metabolism, fatty acid metabolism, nitrogen metabolism, carbon metabolism, and oxidative phosphorylation (Figure 4G). These findings indicated the predominant involvement of fatty acid metabolism, amino acid synthesis, and energy metabolism pathways. Notably, WA demonstrated significantly elevated levels of growth-associated metabolites, including oleoyl ethanolamide, betaine, inosine, docosahexaenoic acid (DHA), PC (38:6), and thymidine, compared to WCC and RCC (*p* < 0.05).

### 3.6. Analysis of Muscle Transcriptomics

**Figure 5:**
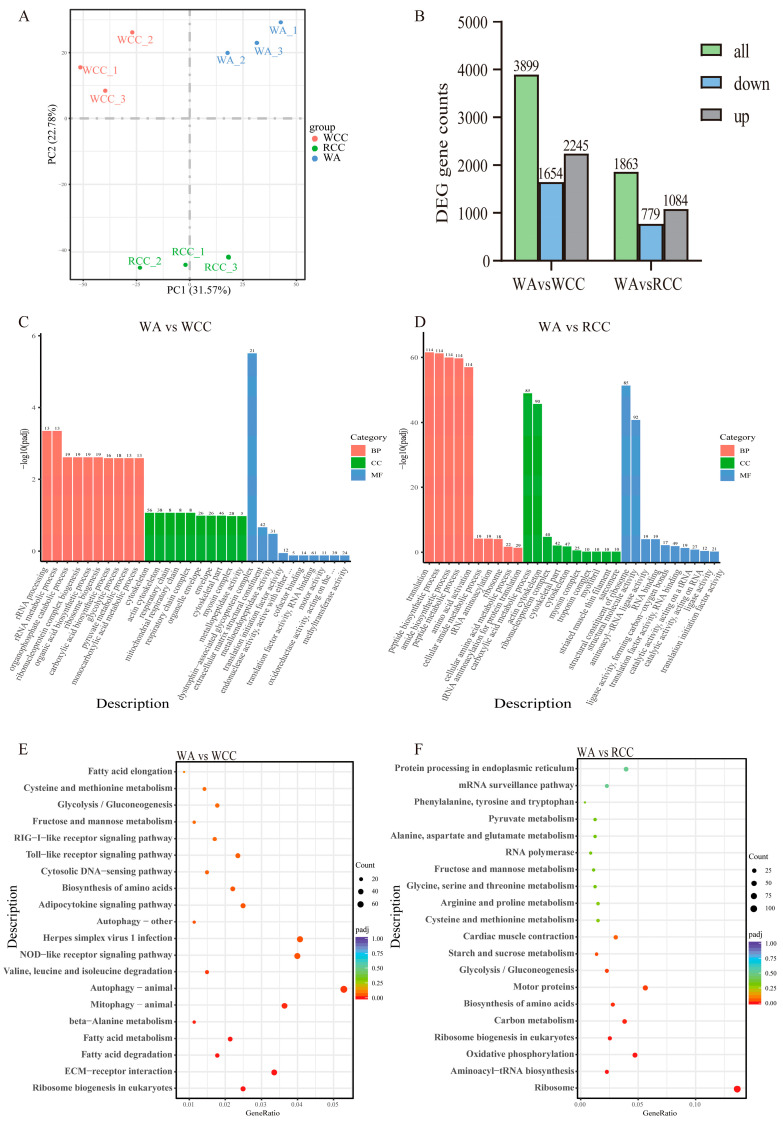
Identification and function enrichment of the DEGs in WA, WCC, and RCC. (**A**) PCA plot. (**B**) The number of DEGs in the two comparison groups of fish: blue represents the downregulated genes; gray represents the upregulated genes. (**C**,**D**) GO enrichment analysis of the DEGs in two comparison groups. (**E**,**F**). KEGG enrichment analysis of the DEGs in two comparison groups.

Muscle transcriptomes from WA, WCC, and RCC were sequenced, yielding 4.36 × 10^8^ clean reads (65.33 GB) (Table S7). We identified 3899 DEGs in WA vs. WCC (2245 upregulated and 1654 downregulated) and 1863 DEGs in WA vs. RCC (1084 upregulated and 779 downregulated) (Figure 5B). GO enrichment analysis of DEGs indicated that the pathways significantly enriched in WA vs. WCC included rRNA processing, rRNA metabolic process, and extracellular matrix structural constituents (Figure 5C), whereas those enriched in WA vs. RCC were translation, peptide biosynthetic process, and amide biosynthetic process (Figure 5D). KEGG enrichment analysis revealed that the pathways significantly enriched in WA vs. WCC included Ribosome biogenesis in eukaryotes, biosynthesis of amino acids, Autophagy—animal, Fatty acid metabolism and Fatty acid degradation (Figure 5E), whereas the pathways enriched in WA vs. RCC were Ribosome, Carbon metabolism, Oxidative phosphorylation, and Biosynthesis of amino acids (Figure 5F). Among these, growth-related pathways included Biosynthesis of amino acids (ko01230), Oxidative phosphorylation (ko00190), and Carbon metabolism (ko01200). These results suggest that WA is mainly associated with lipid metabolism, protein synthesis, and energy metabolism in DEGs. Meanwhile, we identified 15 DEGs associated with growth, including myosin heavy chain (*myh*), alpha skeletal muscle actin (*acta1*), myogenin (*myog*), fructose-bisphosphate aldolase a (*aldoa*), activin a receptor type 2b (*acvr2b*), protein kinase b (*akt*), cysteine-rich protein 61 (*cyr61*), growth hormone receptor (*ghr*), htra serine peptidase 1 (*htra1*), insulin receptor substrate 2 (*irs2*), insulin-like growth factor-binding protein 3 (*igfbp3*), myogenic factor 6 (*myf6*), myogenic differentiation antigen 1 (*myod1*), myostatin b (*mstnb*), and vascular endothelial growth factor a (*vegfa*) (Table 5).

### 3.7. Combined Metabolome and Transcriptome Analysis

**Figure 6:**
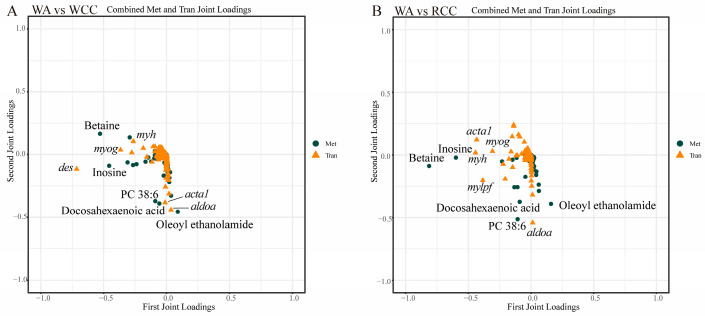
Analysis of DEGs and DMs. (**A**) O2PLS model loading plots for DEGs and DMs in WA vs. WCC. (**B**) O2PLS model loading plots for DEGs and DMs in WA vs. RCC. Labels represent the top 5 genes and metabolites contributing to the O2PLS model.

**Figure 7:**
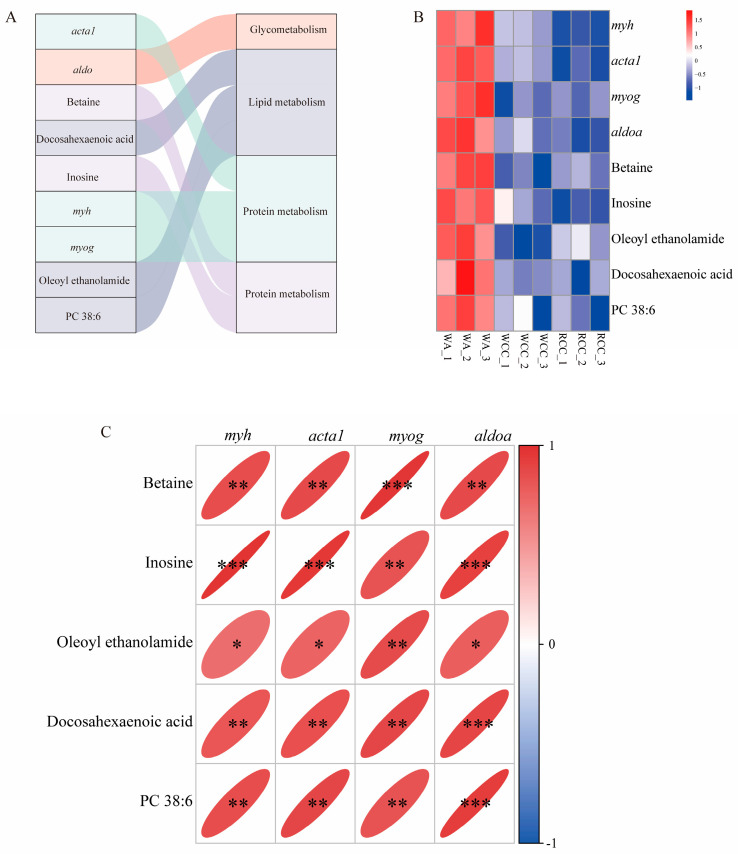
Analysis of the top 5 shared genes and metabolites in WA vs. WCC and WA vs. RCC. (**A**) Metabolic types of the top 5 shared genes and metabolites. (**B**) Heatmap of top 5 shared genes and metabolites. (**C**) Pearson correlation analysis of the top 5 shared genes and metabolites. * Indicates statistical significance (*p* < 0.05), ** *p* < 0.01, *** *p* < 0.001.

O2PLS models were constructed using the DMs and DEGs as databases to further elucidate the key molecular response mechanisms for the rapid growth of WA. The five genes (*myh*, *acta1*, *myog*, *aldoa*, and *desmin* (*des*)) and five metabolites (oleoyl ethanolamide, betaine, inosine, DHA, and PC (38:6)) with the highest contribution scores in the O2PLS model were identified in WA vs. WCC (Figure 6A); the five genes (*myh*, *acta1*, *aldoa*, *myog*, fast skeletal myosin light chain 2 (*mylpf*)) and five metabolites (oleoyl ethanolamide, betaine, inosine, DHA, and PC (38:6)) with the highest contribution scores in the O2PLS model were identified in WA vs. RCC (Figure 6B). Four genes (*myh*, *acta1*, *aldoa*, *myog*) were common to both comparisons, whereas the same five metabolites were identified in both analyses. These genes and metabolites were mainly involved in protein metabolism, glycometabolism, and lipid metabolism (Figure 7A). These genes and metabolites were upregulated in WA compared to that in WCC and RCC (Figure 7B). Pearson correlation analysis between these four genes and the five metabolites showed positive correlations (*r* > 0.8, *p* < 0.05) (Figure 7C).

### 3.8. RT-qPCR Validation

**Figure 8:**
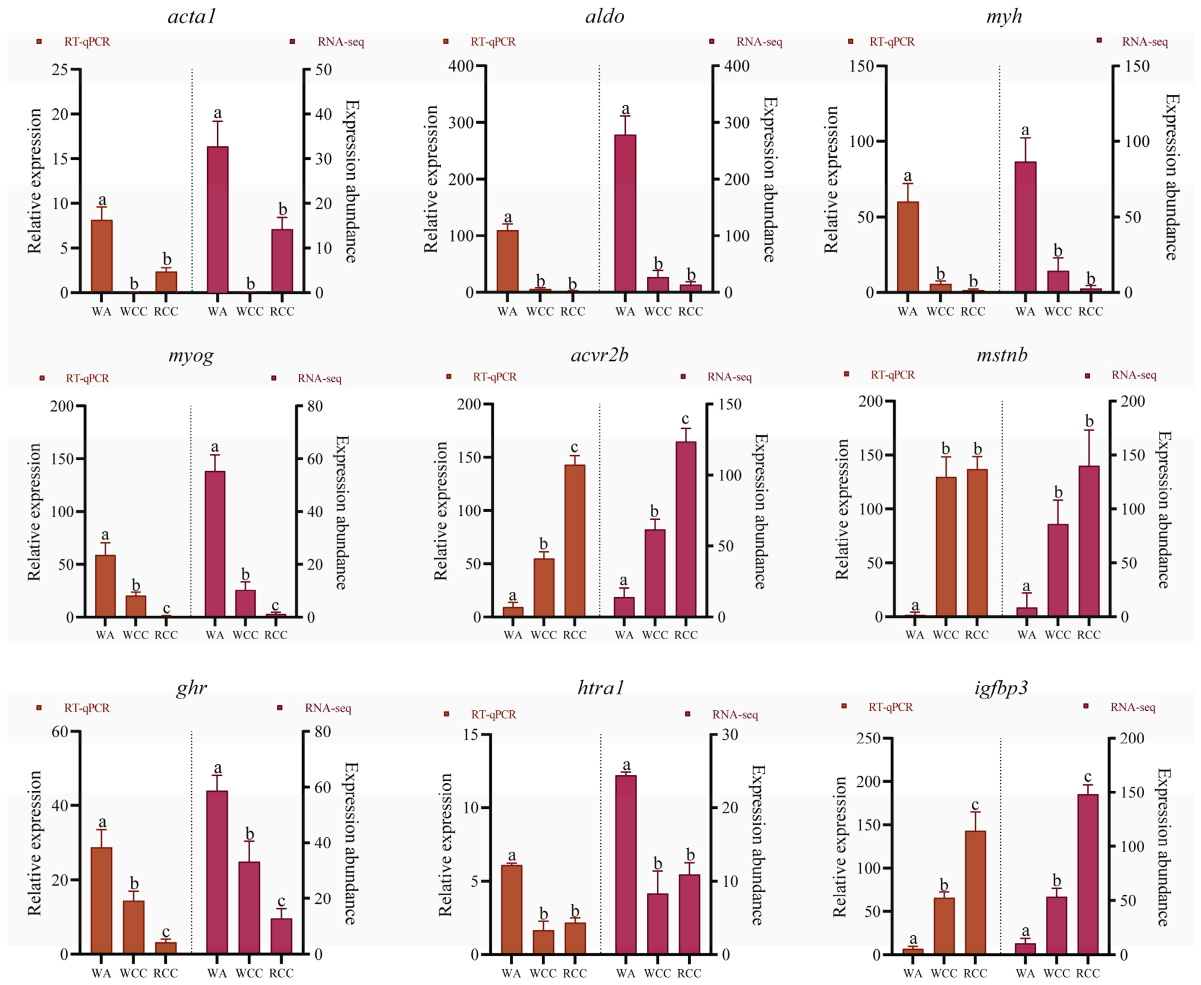

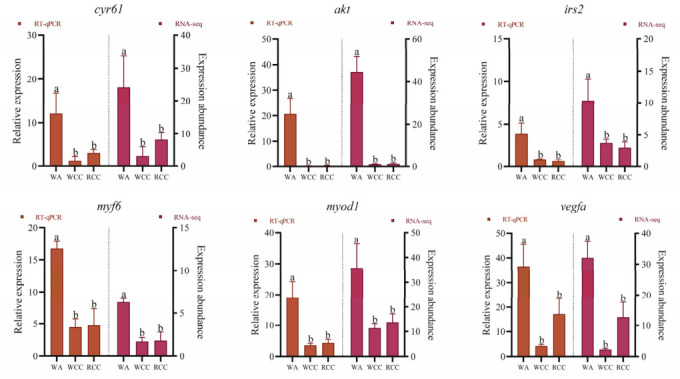
The relative mRNA levels of key growth-related genes in WA, WCC, and RCC were determined by RT-qPCR. Different lowercase letters indicate significant differences (*p* < 0.05).

We validated 15 growth-related DEGs through RT-qPCR analysis. Among these genes, 12 (*acta1*, *aldo*, *myh*, *myog*, *ghr*, *htra1*, *cyr61*, *akt*, *irs2*, *myf6*, *myod1*, *vegfa*) showed significantly higher expression levels in WA than in WCC and RCC. The expression levels of two genes (*acvr2b*, *mstnb*) in WA showed no significant difference compared to 4nAT but were significantly lower than those in WCC, RCC, and WR-II. The expression level of one gene (*igfbp3*) in WA was significantly lower than that in WCC and RCC (Figure 8).

## 4. Discussion

Distant hybridization technology is widely used to breed new aquatic species with fast growth rates, strong resistance, and excellent meat quality [35]. Polyploid hybrid fish are widely used in scientific research and aquaculture. Examples include triploid (3*n* = 124) and tetraploid (4*n* = 148) hybrids resulting from crosses between red crucian carp and blunt snout bream [3], and sterile triploid crucian carp resulting from crosses between white crucian carp and allopolyploid fish, which have become important sources of protein [36]. Among polyploid fish, fast-growing triploids have the broadest range of applications. In several fish breeding programs, triploid production has been successfully achieved through crosses between diploid and tetraploid fish [10,36]. In the present study, we developed a novel triploid fish (WA, 3*n* = 150) using distant hybridization between a diploid hybrid fish (WR-II, 2*n* = 100) and a heterozygous tetraploid fish (4nAT, 4*n* = 200). The hybrid combination showed high fertilization (89.22%) and hatchability (78.71%) rates, indicating the potential feasibility of large-scale fry production. In this study, WA is expected to have reduced reproductive potential due to its triploidy, which may decrease the risk of genetic introgression into wild populations. However, reproductive sterility and ecological impacts require further evaluation [16]. This characteristic confers greater ecological safety on WA compared to fertile hybrids when applied to large-scale aquaculture [19,20]. Although triploids offer ecological safety advantages, they also present trade-offs in traits. For example, whilst triploid yellow croaker exhibits rapid growth, it displays reduced resistance to the bacterium V. alginolyticus and diminished swimming endurance [37]; their application must therefore be tailored to specific circumstances.

Distant hybridization typically induces phenotypic variations in offspring while conferring hybrid vigor, which is characterized by enhanced growth performance, improved disease resistance, and superior muscle nutritional quality [1,38]. For example, WR-F_1_ hybrids displayed distinct gray body coloration and accelerated growth compared with their parental strains [25]; WR-II hybrids demonstrating rapid growth, enhanced stress tolerance, and elevated muscle protein content [25]; and the morphological traits of hybrids between female *Megalobrama amblycephala* and male *Culter alburnus* exhibit hybrid characteristics [10,39]. However, not all triploid fish exhibit a growth advantage; certain triploid fish may experience slowed growth at specific stages, which may be related to genomic instability or an imbalance in energy allocation [8]. Polyploidization may confer advantages such as an increased cell volume and altered metabolic characteristics, but it may also be accompanied by higher energy maintenance costs and an increased risk of oxidative stress [37]. The molecular mechanisms underlying triploid growth may vary across different fish species, and comparative studies across a wider range of species are required in the future. In the present study, both countable and quantifiable WA traits exhibited hybridization characteristics. Notably, WA exhibited a significantly higher WGR and SGR than WCC and RCC (*p* < 0.05), indicating rapid growth. This phenotype may reflect the combined effects of hybrid vigor, polyploidization, and the genetic backgrounds of the parents. In addition, muscle fibers of WA showed significant hypertrophy, and the muscle exhibited higher protein, fat, and amino acid contents compared to that in WCC and RCC. Therefore, the rapid growth of WA may be associated with muscle hypertrophy and elevated protein and fat contents in muscle tissue.

Growth performance is a key economic trait in fish breeding programs and is regulated by multiple genes [40]. Identifying key growth-related genes and metabolites can help elucidate the molecular mechanisms underlying accelerated growth in fish. Muscle metabolite analysis revealed significantly higher levels of oleoyl ethanolamide, betaine, inosine, DHA, thymidine, and PC (38:6) in WA compared to that in WCC and RCC. According to reports, oleoyl ethanolamide has been reported to regulate fatty acid metabolism through PPAR-related pathways, thereby optimizing energy metabolism and growth efficiency [41,42,43], while betaine serves as an effective growth promoter and has been successfully applied as a fish feed additive [44,45,46]. As an ATP metabolic intermediate, inosine regulates lipid metabolism and promotes development through AMPK pathway-mediated fatty acid oxidation [47,48,49,50], and DHA is an important long-chain polyunsaturated fatty acid involved in membrane function, metabolism, and physiological development [51]. The elevated concentrations of these key metabolites in WA muscle tissue likely contributed to its superior growth performance compared to that of WCC and RCC. Furthermore, comparative transcriptomic analysis of muscle tissue identified 15 DEGs associated with growth regulation. The expression changes of these genes were associated with the rapid growth phenotype of WA. Among these, *mstnb*, *acvr2b*, *myod1*, *myog*, *myh*, *acta1*, and *myf6* are related to muscle development and differentiation and may contribute to the regulation of muscle growth [52,53,54,55,56,57,58]; *akt*, *irs2*, *aldoa*, *ghr*, and *igfbp3* promote muscle growth by mediating the fish insulin, IGF-1, and mTOR pathways [59,60,61,62]; and *cyr61*, *vegfa*, and *htra1* are growth factors that positively regulate growth and development [63,64,65]. A combined metabolomics and transcriptomics analysis can provide a basis for understanding the rapid growth characteristics of WA. O2PLS modeling identified consistent key metabolites (oleoyl ethanolamide, betaine, inosine, DHA, and PC (38:6)) and growth-related genes (*acta1*, *aldoa*, *myog*, *myh*) in WA vs. WCC and WA vs. RCC. Pearson’s correlation analysis showed a positive correlation between the four genes and the five metabolites (Figure 7C). We hypothesized that WA muscle hypertrophy may be due to the high expression of these genes, which, in turn, promotes WA muscle growth and development. These genes and metabolites may contribute to the metabolic and molecular processes associated with accelerated growth in WA. The conclusions of this study are primarily based on multi-omics correlation analyses; the proposed gene–metabolite–growth regulatory network requires further functional validation. Although the integrated O2PLS analysis and RT-qPCR validation provide multidimensional evidence for our inferences, a direct causal relationship has not yet been established. In future studies, key candidate genes could be knocked out or overexpressed using CRISPR/Cas9 to validate their regulatory roles in muscle growth and development. Concurrently, the expression of relevant proteins could be assessed via Western blot analysis. Furthermore, omics analyses can be conducted on WA in combination with its parental lines (WR-II and 4nAT) to further elucidate the relative contributions of hybridization and polyploidization. Collectively, these findings suggest that the superior growth performance of WA is associated with coordinated changes in genes involved in myogenesis, protein biosynthesis, and growth-related signaling pathways.

## 5. Conclusions

In this study, we obtained a novel allotriploid fish (WA; 3*n* = 150) using distant hybridization. WA has 150 chromosomes, and its morphological characteristics show intermediate features between the parental strains. In addition, WA exhibits enhanced growth performance, and WA muscle fibers show significant hypertrophy. Integrated transcriptomic and metabolomic analyses indicated that genes associated with muscle growth, development and energy metabolism (*acta1*, *aldoa*, *myog*, and *myh*) were significantly upregulated, and that the abundance of related metabolites (oleoyl ethanolamide, betaine, inosine, DHA, and PC (38:6)) was significantly elevated in WA. In future studies, we plan to validate gene functions through gene editing techniques and explore the functional roles of these genes in regulating growth-related processes. The enhanced growth phenotype of WA is likely associated with coordinated changes in muscle development and metabolic pathways, together with the effects of hybridization and polyploidization. This study provides candidate molecular markers associated with growth performance in triploid fish and contributes to understanding the molecular basis of enhanced growth phenotypes.

## Figures and Tables

**Table 1:** The ratios of measurable traits of WCC, RCC, WR-II, 4nAT, and WA.

Fish Type	WL/BL	BL/BH	BL/HL	HL/HH	CPL/CPH
WCC	1.24 ± 0.03	2.31 ± 0.15 ^cde^	3.78 ± 0.40 ^cde^	1.16 ± 0.05 ^cde^	0.83 ± 0.05 ^cde^
RCC	1.23 ± 0.02	2.24 ± 0.12 ^cde^	3.74 ± 0.35 ^cde^	1.17 ± 0.06 ^cde^	0.81 ± 0.06 ^cde^
WR-II	1.20 ± 0.01	1.97 ± 0.10 ^abde^	4.03 ± 0.34 ^ab^	1.03 ± 0.08 ^ab^	0.88 ± 0.11 ^de^
4nAT	1.25 ± 0.05	2.52 ± 0.24 ^abc^	4.21 ± 0.36 ^ab^	1.04 ± 0.03 ^ab^	1.05 ± 0.09 ^abc^
WA	1.26 ± 0.02	2.41 ± 0.19 ^abc^	4.09 ± 0.31 ^ab^	1.08 ± 0.09 ^ab^	1.07 ± 0.08 ^abc^

Notes: Values are means ± SE, *n* = 100. The abbreviations in the table represent: WL/BL, whole length/body length. BL/BH, body length/body height. BL/HL, body length/head length. HL/HH, head length/head height. CPL/CPH, caudal peduncle length/caudal peduncle height. ^a, b, c, d^ and ^e^ represent significant differences from WCC, RCC, WR-II, 4nAT, and WA, respectively, within the same column of data (*p* < 0.05).

**Table 2:** The morphological assessment of WCC, RCC, WR-II, 4nAT, and WA.

Fish Type	Lateral Line Scales	Upper Lateral Line Scales	Lower Lateral Line Scales	Dorsal Fins	Abdominal Fins	Anal Fins
WCC	32–34	6–8	5–7	III + 18–20	8–10	III + 6–7
RCC	28–30	5–6	6–7	III + 18–20	8–9	III + 6–7
WR-II	30–32	6–7	6–7	III + 18–21	8–9	III + 6–7
4nAT	30–34	5–7	5–8	III + 16–19	6–8	III + 5–7
WA	30–31	5–6	6–7	III + 17–20	8–9	III + 6–7

Notes: Values are means ± SE, *n* = 100. “III” represents hard fin rays.

**Table 3:** The ratios of measurable traits in WCC, RCC, and WA.

Fish Type	Moisture (g/100 g)	Fat (g/100 g)	Protein (g/100 g)	Ash (g/100 g)	Carbohydrates (g/100 g)	Energy Value (kJ/100 g)
WCC	75.5 ^b^	3.6 ^b^	14.8 ^b^	1.7 ^b^	79.9	1721.87
RCC	74.5 ^b^	3.7 ^b^	15.8 ^b^	2.2 ^a^	78.3	1715.59
WA	71.7 ^a^	4.5 ^a^	17.7 ^a^	1.6 ^b^	76.2	1742.38

Notes: The data in the table are mean values. *n* = 10. Different lowercase letters indicate significant differences within the same column of data (*p* < 0.05).

**Table 4:** The ratios of measurable traits in WA, WCC, and RCC.

Amino Acid	WA	WCC	RCC
Asp	1.58	1.18	1.38
Thr	0.69	0.53	0.61
Ser	0.61	0.46	0.54
Glu	2.14	1.63	1.88
Gly	0.71	0.54	0.60
Ala	0.93	0.71	0.81
Cys	0.12	0.086	0.11
Val	0.78	0.59	0.70
Met	0.31	0.22	0.26
IIe	0.67	0.52	0.60
Leu	1.24	0.95	1.09
Tyr	0.49	0.38	0.44
Phe	0.69	0.52	0.62
Lys	1.51	1.19	1.34
His	0.43	0.30	0.37
Arg	0.88	0.69	0.81
Pro	0.47	0.39	0.43
∑ DAA	5.36 ^bc^	4.06 ^a^	4.67 ^a^
∑ EAA	5.89 ^bc^	4.52 ^ac^	5.22 ^ab^
∑ TAA	14.25 ^bc^	10.89 ^a^	12.59 ^a^

Notes: The data in the table are mean values. Σ DAA: Total flavor-related amino acids; Σ EAA: Total essential amino acids; Σ TAA: Total amino acids. ^a, b^ and ^c^ represent significant differences from WA, WCC, and RCC, respectively, within the same row of data (*p* < 0.05).

**Table 5:** Genes associated with growth shared among the sets of WA DEGs.

Gene Symbol	Gene Description	log2 (WA/WCC)	log2 (WA/RCC)
*mstnb*	myostatin b	−3.64	−2.61
*igfbp3*	insulin-like growth factor-binding protein 3	−2.84	−3.42
*acvr2b*	activin receptor type-2b	−2.41	−3.86
*vegfa*	vascular endothelial growth factor a	1.75	2.36
*myod1*	myogenic differentiation antigen 1	1.78	1.23
*irs2*	insulin receptor substrate 2	1.81	1.54
*myf6*	myogenic factor 6	1.82	1.49
*cyr61*	cysteine rich protein 61	2.24	2.51
*akt*	protein kinase b	2.32	2.04
*aldoa*	fructose-bisphosphate aldolase a	2.54	1.89
*ghr*	growth hormone receptor	3.61	4.06
*myog*	myogenin	3.65	3.49
*htra1*	htra serine peptidase 1	4.48	5.32
*myh*	myosin heavy chain	5.32	2.31
*acta1*	alpha skeletal muscle	7.01	1.28

## Data Availability

The complete clean reads for these libraries have been uploaded to the NCBI Sequence Read Archive site (http://www.ncbi.nlm.nih.gov/sra/ (accessed on 10 November 2025); Accession Nos. SRR34377147, SRR34377148, SRR34377149, SRR34377150, SRR34377151, SRR34377152, SRR34377153, SRR34377154 and SRR34377155).

## Supplementary Materials

The following supporting information can be downloaded at: https://www.mdpi.com/article/10.3390/ani16152341/s1, Table S1: Specific parameters for LC-MS; Table S2: The primers of quantitative real-time PCR; Table S3: The fertilization rate and hatching rate of WA; Table S4: Mean DNA content of WR-II, 4nAT, and WA; Table S5: Chromosome number at mitotic metaphase in WA; Table S6: Average body weight, WGR and SGR at different months of age in WA, WCC and RCC (g); Table S7: Basic information of the muscle transcriptomes of WA, WCC, and RCC.
