# Supplementary material for: Transcriptomic and Metabolomic Insights into Growth Performance of a Novel Allotriploid Fish Derived from Carassius cuvieri, Carassius auratus red var., and Cyprinus carpio
Source: Animals (Basel). 2026 Aug 1;16(15):2341. doi: 10.3390/ani16152341 (PMC13463577; doi:10.3390/ani16152341)
Supplement: Supplementary file 1 [file animals-16-02341-s001.zip › animals-4392194-supplementary.pdf]

Table S1. Specific parameters for LC-MS.

Liquid chromatography conditions: Column: Waters ACQUITY UPLC HSS T3 (2.1 × 100 mm, 1.8 μm); Column temperature: 40 °C; Mobile phase A: 0.1% formic acid in water; Mobile phase B: 0.1% formic acid in acetonitrile; Flow rate: 0.35 mL/min; Injection volume: 2 μL. Gradient programmed: 0–2 min, 5% B; 2–12 min, 5%–95% B; 12–14 min, 95% B; 14–14.1 min, 95%–5% B; 14.1–16 min, equilibration at 5% B.

Mass spectrometry conditions: Instrument: Thermo Fisher Q Exactive Orbitrap;

Quality control: A pooled QC sample was prepared by mixing equal volumes from all samples. One QC sample was inserted after every 10 analytical samples, with five QC samples run at the start and end of the sequence to allow the system to equilibrate. Characteristic peaks in the QC samples with an RSD (relative standard deviation) < 30% were retained. Peak alignment, retention time correction and feature extraction were performed using XCMS software.

Table S2. The primers of quantitative real-time PCR.

Gene	primer sequence (5′–3′)	primer sequence (5′–3′)
β-actin	F: TGCTGTCCCTGTATGCCTCT	R: TTTGATGTCACGCACGATTT
mstnb	F: CGGTTCAGTGGGTCATGGAGA	R: GCCTGTTTGAGTCGGAGTTTGC
igfbp3	F: CCAATGGTACCAGGGCAACT	R: TGGACTCTATTGCCATCCTCG
acvr2b	F: TGGTTTACTCTCTGCTGCCCGT	R: AGATGGAGCCACAGAAGCACAC
vegfa	F: CGTTGGCGAGTCTGTCTGTGT	R: TCCGATTGATTGTGGATTTATGG
myod1	F: GACGCTTTCGAGACCCTCAA	R: GTATTCCGTGCGTCAGCATTC
irs2	F: AGACTGGATGAGCCTGAG	R: CTGTATGATGACGATGATGAC
myf6	F: GGTCATCGAGAAGAAGCC	R: GAAGGAAATGTGAGGTGGAT
cyr61	F: TACCCCTACAAGCCCAAAGACT	R: TGACCCGAGATGAAACGCCC
akt	F: GAGAAAGCCAGCGGCACATA	R: CCATTCACATACTCCATAACAAAAC
aldoa	F: GCAACTTATTAACGTGGTCG	R: TGAGGCATCTTGACTCAGTT
ghr	F: CGAGGACTTGGCTACCCTTC	R: TCTGATGTCTGTTGGACGCC
myog	F: AGACCAATCCCTACTTCTTCGCAG	R: CCATCACCACACAAGCCCACC
htra1	F: GCCAACAAACACAGGGTGAAG	R: CCACAAACTCGCCTGGTCTC
myh	F: GTGCAAAACTGACCTCAATAA	R: GAATGTTTTACAGCCCACAAG
acta1	F: CTTGCGTGCTCTTATTTG	R: CTTGCGTGCTCTTATTTG

Table S3. The fertilization rate and hatching rate of WA.

Fish Type	Fertilization rate	Hatching rate	Hatching time	Sailing time	Hatching water temperature
WA	89.22%	78.71%	48–63h	72–87h	22–24℃

Table S4. Mean DNA content of WR-Ⅱ, 4nAT, and WA.

Species/hybrids	Mean DNA content	Ratio	
		Observed	Expected
WR-Ⅱ	98.65		
4nAT	201.53		
WA	151.18	WA/WR-Ⅱ=1.53[a]	1.5

Note: [a] The observed ratio is not significantly different ($P > 0.05$) from the expected ratio.

Table S5. Chromosome number at mitotic metaphase in WA.

Fish type	Number of samples	Number of metaphase spreads	Chromosome number[a]		
			150	149	148
WA	10	100	91	7	2

Table S6. Average body weight, WGR and SGR at different months of age in WA, WCC and RCC. (g)

Months	WCC	RCC	WA
2 months	51.30 ± 1.81^{c}	42.97 ± 1.72^{c}	61.40 ± 2.07^{ab}
4 months	92.73 ± 2.43^{c}	81.30 ± 2.03^{c}	102.53 ± 2.26^{ab}
6 months	126.03 ± 2.81^{c}	112.53 ± 2.72^{c}	142.77 ± 7.01^{ab}
8 months	162.93 ± 3.56^{c}	143.8 ± 3.67^{c}	182.30 ± 3.79^{ab}
10 months	194.47 ± 4.35^{c}	183.83 ± 4.12^{c}	238.31 ± 4.91^{ab}
12 months	213.33 ± 4.76^{c}	193.73 ± 5.89^{c}	321.32 ± 19.91^{ab}
WGR (%)	4023.13^{c}	3643.75^{c}	6100.13^{ab}
SGR (%/d)	1.03^{c}	1.00^{c}	1.15^{ab}

Notes: Values are means ± SE. $^{a, b}$ and ^{c} represent significant difference from WCC, RCC and WA, respectively, within the same row of data ($P < 0.05$).

Table S7. Basic information of the muscle transcriptomes of WA, WCC, and RCC.

sample	raw Reads	clean Reads	clean Bases (GB)	Q20 (%)	GC (%)	exon ratio (%)
WA_1	44866664	43951000	6.59	98.22	48.86	69.77
WA_2	46580376	45696748	6.85	98.34	49.23	72.19
WA_3	46547554	45475330	6.82	98.45	48.66	68.99
WCC_1	52447152	51578724	7.74	98.3	48.57	69.27
WCC_2	48737584	47974478	7.2	98.29	48.89	69.06
WCC_3	52565840	51739902	7.76	98.24	48.49	68.10
RCC_1	47670914	46890626	7.03	98.25	48.06	60.33
RCC_2	52620182	51665494	7.75	98.32	48.69	66.45
RCC_3	51822116	50579014	7.59	98.15	47.8	59.23
